# The prognostic and diagnostic significance of the neutrophil-to-lymphocyte ratio in hepatocellular carcinoma: a prospective controlled study

**DOI:** 10.1038/s41416-021-01445-3

**Published:** 2021-06-14

**Authors:** Philip J. Johnson, Sofi Dhanaraj, Sarah Berhane, Laura Bonnett, Yuk Ting Ma

**Affiliations:** 1 Department of Molecular and Clinical Cancer Medicine, University of Liverpool, Liverpool, UK; 2 Liver Services, University Hospitals NHS Foundation Trust, Birmingham, UK; 3 Institute of Applied Health Research, University of Birmingham, Birmingham, UK; 4 Department of Health Data Science, University of Liverpool, Liverpool, UK; 5 Institute of Immunology and Immunotherapy, University of Birmingham, Birmingham, UK

**Keywords:** Hepatocellular carcinoma, Hepatocellular carcinoma

## Abstract

**Background:**

The neutrophil–lymphocyte ratio (NLR), a presumed measure of the balance between neutrophil-associated pro-tumour inflammation and lymphocyte-dependent antitumour immune function, has been suggested as a prognostic factor for several cancers, including hepatocellular carcinoma (HCC).

**Methods:**

In this study, a prospectively accrued cohort of 781 patients (493 HCC and 288 chronic liver disease (CLD) without HCC) were followed-up for more than 6 years. NLR levels between HCC and CLD patients were compared, and the effect of baseline NLR on overall survival amongst HCC patients was assessed via multivariable Cox regression analysis.

**Results:**

On entry into the study (‘baseline’), there was no clinically significant difference in the NLR values between CLD and HCC patients. Amongst HCC patients, NLR levels closest to last visit/death were significantly higher compared to baseline. Multivariable Cox regression analysis showed that NLR was an independent prognostic factor, even after adjustment for the HCC stage.

**Conclusion:**

NLR is a significant independent factor influencing survival in HCC patients, hence offering an additional dimension in prognostic models.

## Introduction

The main prognostic clinical features for hepatocellular carcinoma (HCC) are now well-documented and include tumour size and number, presence of vascular invasion, serum alpha-fetoprotein (AFP) levels and performance status^1^ lymphocyte-to-neutrophil ratio (NLR), initially proposed as an index of systemic inflammation in the critically ill^2^ has recently attracted interest in the field of cancer in general^3–5^ and specifically in HCC.^6,7^ Very few of the studies have been based on prospectively collected datasets, and the absolute value of NLR compared to that of patients with liver disease without HCC has received very little attention. Furthermore, follow-up has usually been short and confounding factors that might have inappropriately indicated a prognostic or diagnostic role for NLR have not been examined. The recent observation that NLR was a significant predictor of response to sorafenib in patients with advanced HCC has expanded the role of NLR in HCC.^8^

## Patients and methods

This study comprised 781 patients, 493 with HCC diagnosed according to international guidelines and a control group of 288 patients with chronic liver disease (CLD) alone. All patients were recruited from Queen Elizabeth Hospital (Birmingham, UK) between 2007 and 2012. Those with HCC were from the HCC clinic; those with CLD were from hepatology out-patient clinics from where we accepted locally defined Child–Pugh score (CPS) and cirrhosis status, acknowledging that strictly speaking CPS should not be applied in the cases where cirrhosis was not present (Supplementary Table 1). The study was approved by the South Birmingham Ethics Committee (CARMS-13594). HCC treatment was decided upon at a multidisciplinary team meeting, broadly in line with BCLC recommendations, and laboratory results were those measured at the time of study recruitment. However, we did not study directly the changes in NLR according to specific treatments, and this is acknowledged as a limitation of the study.

Evidence suggests that a low NLR is associated with a better prognosis. Therefore, we considered the possibility that there may be a significant difference, with diagnostic potential, between NLR in HCC and a relevant control population. The reference range for NLR was taken from Forget et al.^9^ For the analysis conducted here, ‘baseline’ refers to the values of the blood results taken at the time of entry into the study. In the case of patients with HCC, this was before any treatment. Last NLR prior to death or last follow-up was also taken for HCC patients. Other variables recorded were age, sex, aetiology, Child–Pugh grade, albumin (g/l), bilirubin (μmol/l), international normalised ratio (INR), creatinine (µmol/l), haemoglobin (g/l), total white blood count (WBC, ×10^9^/l), neutrophil count (×10^9^/l), lymphocyte count (×10^9^/l), platelet count (×10^9^/l), alpha-fetoprotein (AFP), tumour number, tumour size (cm), presence of vascular invasion (yes/no), extrahepatic spread (EHS) and Barcelona Clinic Liver Cancer (BCLC) staging. The last five listed variables were related to HCC only.

### Statistical methods

Analysis was carried out using Stata/SE 16 (StataCorp, Texas, USA). Continuous variables were reported as mean (with standard deviation [SD]) or median (with interquartile range (IQR)), the latter for variables with skewed distributions. Categorical variables were presented as percentages. For skewed variables, different transformations were explored—cubic, square, identity, square root, log, 1/(square root), inverse, 1/square, 1/cubic—and the resulting distributions visually examined via q–q (quantile–quantile) plots. The transformation that has a distribution closest to normal was selected. Further details of the statistical methodology are presented in the Supplementary Data section.

## Results

### NLR levels in CLD versus HCC patients—the diagnostic role of NLR

At baseline, NLR was higher in the HCC cohort compared to the control subjects (2.79 vs 2). Although this difference was statistically significant (*P* < 0.0001), it was unlikely to be of any clinical significance. However, when baseline NLR of the HCC patients was compared to the levels taken closest to last visit or death, it became apparent that NLR rose significantly (from 2.79 to 4.59, *P* < 0.0001) (Supplementary Fig. 1). The corresponding AUROC estimate was 0.65 (95% CI 0.62, 0.69), showing that NLR is only very weakly diagnostic.

### Prognostic analysis

Median overall survival was 12.60 months (95% C.I. 10.23, 14.51) for HCC patients, whereas this was not reached for CLD patients (Supplementary Fig. 2). Univariable Cox regressions have shown that NLR (log-transformed) is a prognostic factor in HCC patients (*P* < 0.0001) but not in CLD patients (*P* = 0.200) (Supplementary Table 2).

**Fig. 1:**
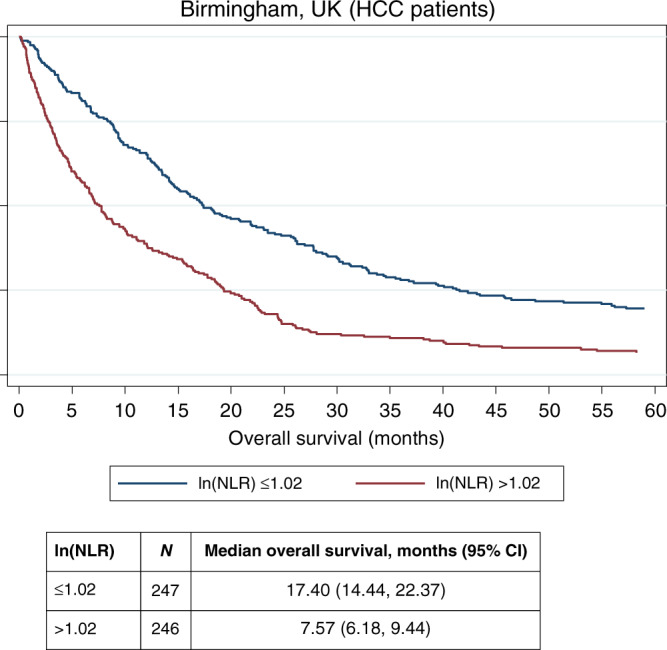
Kaplan–Meier survival curves according to median ln(NLR) among HCC patients. NLR categories generated by applying cut-off at the median.

In a Cox regression model, NLR remains one of the independent factors influencing overall survival along with age, bilirubin, AFP, haemoglobin, WBC and BCLC stage (Supplementary Table 3). Survival among those with levels below the median (*n* = 247) had an overall survival of 17.40 months (95% C.I. 14.44, 22.37) and those with NLR greater than the median (*n* = 246) survived 7.57 months (95% C.I. 6.18, 9.44) (*P* < 0.0001) (Fig. 1).

## Discussion

The great majority of previous studies have been retrospective, with all the attendant limitations, and have largely been focused on specific stages of the disease. Here, we confirm that within a single large referral practice over a recent period, NLR is indeed a statistically significant independent prognostic (but not diagnostic) factor. Although the absolute survival figures varied according to disease stage (and hence treatment), within each disease stage NLR remained an independent prognostic factor.

The optimal ‘cut-off point’ to use in a clinical setting has been the source of extensive discussion.^10^ The median value has been widely advanced and our more formal statistical analysis suggests that this is, indeed, close to optimal. The difference in survival above and below the median of 7 months vs 18 months is clearly clinically significant. However, it is difficult to conceive of a situation in which NLR would be useful in a clinical setting since; as shown here it will only be one of several interlinked factors influencing survival. We, therefore, show that it is entirely feasible to build a simple prognostic model for HCC, which takes into account all defined prognostic factors and includes NLR. This may be particularly important as an additional dimension to the three current dimensions - performance status, liver function and tumour characteristics. The recent analysis of the SHARP and AP placebo-controlled sorafenib trials, which showed that NLR was a predictive factor for benefit from Sorafenib, support this contention^8^ and we can show that adding NLR to our prognostic model (PROSASH) for sorafenib-treated patients does indeed improve performance.

## Supplementary information

The prognostic and diagnostic significance of the neutrophil to lymphocyte ratio in hepatocellular carcinoma: A prospective controlled study

## Data Availability

All reasonable requests will be considered on application to the corresponding author and subject to review by the Ethics Committee.
