# Supplementary material for: The prognostic and diagnostic significance of the neutrophil-to-lymphocyte ratio in hepatocellular carcinoma: a prospective controlled study
Source: Br J Cancer. 2021 Jun 14;125(5):714–6. doi: 10.1038/s41416-021-01445-3 (PMC8405698; doi:10.1038/s41416-021-01445-3)
Supplement: Supplementary file 1 — The prognostic and diagnostic significance of the neutrophil to lymphocyte ratio in hepatocellular carcinoma: A prospective controlled study [file 41416_2021_1445_MOESM1_ESM.docx]

**The prognostic and diagnostic significance of the neutrophil to lymphocyte ratio in hepatocellular carcinoma: A prospective controlled study by PJ Johnson et al**

**SUPPLEMENTARY MATERIAL**

**SUPPLEMENTARY STATISTICAL METHODOLOGY**

For continuous variables, differences between the CLD and HCC groups were tested using the two-sample t-test or Wilcoxon rank-sum test (the latter when normal distribution could not be obtained following transformation). Categorical variables were compared using Pearson’s chi-squared test and Fisher’s exact test (the latter when patients numbers in one of the subcategories was <5).

*NLR levels in CLD versus HCC patients*

NLR levels between CLD and HCC patients were compared at baseline using the two-sample t-test (see above). Additionally, the distribution of NLR in the two patient groups at baseline and at closest to last visit or death was visualised and compared using boxplots. This was performed only using patients with data available at both time points. Paired t-test was performed to compare changes in NLR at the two time points for each patient group.

*Diagnostic analysis*

The ability of NLR to classify between CLD and HCC patients was assessed by fitting a univariable logistic regression, plotting a receiver operating characteristic (ROC) curve, and producing an area under ROC (AUROC) estimate.

*Prognostic analysis*

Overall survival (OS) was calculated from the date of blood taken until the date of death. Patients who were still alive were censored at date of last follow-up. Survival according to CLD and HCC groups was plotted using the Kaplan-Meier (KM) method. Survival distribution between groups was compared using the log-rank test.

Univariable Cox regression analysis was performed to examine the prognostic influence of NLR in the CLD and HCC groups separately. Using just the HCC patients, univariable Cox regression analysis was carried out using the following parameters: age, sex, aetiology, albumin, bilirubin, INR, creatinine, haemoglobin, WBC, NLR, platelets, AFP and BCLC stage. In order to investigate if NLR remains a prognostic factor even after adjusting for other variables, a multivariable model was built using backward selection of the same variables significant at the 10% level. Tumour number and size, vascular invasion, Child-Pugh grade and EHS were not included in the analysis as these are already components of the BCLC staging. The proportional hazards assumption of the model was tested by examining the plots of scaled Schoenfeld residuals against time for each variable.

Finally, KM survival curves according to NLR categories generated by applying cut-off at the median (the most widely used cut-off value) was plotted for the HCC group to display the prognostic stratification of NLR.

**SUPPLEMENTARY FIGURE 1**

**SUPPLEMENTARY FIGURE 2**

| **Supplementary Table 1:** Patient demographics | | | |
| --- | --- | --- | --- |
| **Variables** | **CLD (N=288)** | **HCC (N=493)** | **p-value (CLD vs HCC)** |
| **Age, years** | 52.39 (44.29, 60.79), n=288 | 65.54 (58.67, 73.06), n=493 | <0.0001 |
| **Male, n(%)** | 182 (63.19), n=288 | 394 (79.92), n=493 | <0.0001 |
|  |  |  |  |
| **Aetiology, n(%)** | n=288 | n=493 | <0.0001 |
| Hepatitis C | 94 (32.64) | 93 (18.86) |  |
| Hepatitis B | 57 (19.79) | 42 (8.52) |  |
| Alcohol | 47 (16.32) | 134 (27.18) |  |
| Other | 90 (31.25) | 224 (45.44) |  |
|  |  |  |  |
| **Child-Pugh grade, n(%)** | n=287 | n=486 | <0.0001 |
| A | 265 (92.33) | 358 (73.66) |  |
| B | 21 (7.32) | 106 (21.81) |  |
| C | 1 (0.35) | 22 (4.53) |  |
|  |  |  |  |
| **Albumin, g/l** | 44 (42, 46), n=288 | 39 (34, 43), n=493 | <0.0001 |
| **Bilirubin, µmol/l** | 11 (8, 17), n=288 | 16 (10, 30), n=492 | <0.0001 |
| **International normalised ratio (INR)** | 1.00 (1.00, 1.10), n=284 | 1.10 (1.00, 1.30), n=485 | <0.0001 |
| **Creatinine, µmol/l** | 72 (63, 85), n=288 | 85 (69, 104), n=491 | <0.0001 |
| **Haemoglobin, g/l** | 13.90 (12.60, 15.10), n=288 | 12.90 (11.60, 14.20), n=492 | <0.0001 |
| **Total white blood cell (WBC) count, x10^9^/l** | 5.60 (4.50, 7.10), n=288 | 6.20 (4.60, 8.00), n=493 | 0.0022 |
| **Neutrophil count, x10^9^/l** | 3.20 (2.40, 4.40), n=288 | 3.80 (2.80, 5.30), n=493 | <0.0001 |
| **Lymphocyte count, x10^9^/l** | 1.60 (1.20, 2.10), n=288 | 1.30 (1.00, 1.80), n=493 | <0.0001 |
| **Platelets, x10^9^/l** | 202 (135, 250), n=288 | 156 (100, 239), n=491 | 0.0003 |
| **Neutrophil-to-lymphocyte ratio (NLR)** | 2.00 (1.44, 3.09), n=288 | 2.79 (1.89, 4.18), n=493 | <0.0001 |
| **Alpha-fetoprotein (AFP), ng/ml** | 2.80 (2.10, 4.65), n=288 | 33.90 (6.00, 633.00), n=479 | <0.0001 |
| **Solitary tumours n(%)** | NA | 253 (52.82), n=479 | NA |
| **Tumour size, cm** | NA | 4.60 (3.00, 8.00), n=436 | NA |
| **Vascular invasion, n(%)** | NA | 118 (24.74), n=477 | NA |
| **Extra-hepatic disease (EHD), n(%)** | NA | 46 (9.45), N=487 | NA |
| **Cirrhosis** | 77 | 88 |  |
| **BCLC** |  | n=470 |  |
| 0/A | NA | 48 (10.21) | NA |
| B | NA | 40 (8.51) | NA |
| C | NA | 344 (73.19) | NA |
| D | NA | 38 (8.09) | NA |
| **Treatment (%)** |  |  |  |
| **Potentially curative**  **Transplantation ;Resection : Ablation** | NA | 3; 3;12 |  |
| **Palliative**  **TACE/TAE ; Systemic therapy** | NA | 40:24 |  |
| **Best supportive care** | NA | 15 |  |
| **Not known** | NA | 3 |  |
|  |  |  |  |
| **Death, n(%)** | 40 (13.89), n=288 | 442 (89.66), n=493 | <0.0001 |
| **Median follow-up time, months (95% CI)** | 75.76 (74.01, 79.44), n=288 | 97.27 (79.93, 105.95), n=493 | <0.0001 |
| **Median overall survival, months (95% CI)** | Not reached, n=288 | 12.60 (10.23, 14.51), n=493 | <0.0001 |
| Continous variables reported as median and interquartile range, categorical variables as counts and percentages. Abbreviations: CI, confidence intervals; NA, not applicable. | | | |

| **Supplementary Table 2:** Univariable cox regression analysis (HCC patients) | | |
| --- | --- | --- |
| **Variables** | **Hazard Ratio (95% CI)** | **p-value** |
| **Age, years** | 1.02 (1.01, 1.03) | <0.0001 |
|  |  |  |
| **Sex** |  |  |
| Female | 1 |  |
| Male | 0.87 (0.69, 1.10) | 0.242 |
|  |  |  |
| **ln(bilirubin, µmol/l)** | 1.22 (1.08, 1.38) | 0.001 |
| **1/(INR^3^)** | 0.76 (0.55, 1.05) | 0.093 |
| **Albumin, g/l** | 0.96 (0.94, 0.97) | <0.0001 |
| **ln(creatinine, µmol/l)** | 1.08 (0.86, 1.37) | 0.507 |
| **ln(AFP, ng/ml)** | 1.16 (1.13, 1.20) | <0.0001 |
| **Haemoglobin, g/l** | 0.84 (0.80, 0.89) | <0.0001 |
| **ln(WBC, x10^9^/l)** | 2.07 (1.63, 2.64) | <0.0001 |
| **ln(Platelets, x10^9^/l)** | 1.47 (1.26, 1.72) | <0.0001 |
|  |  |  |
| **Aetiology** |  |  |
| Hepatitis C | 1 |  |
| Hepatitis B | 0.94 (0.63, 1.42) | 0.776 |
| Alcohol | 1.11 (0.83, 1.47) | 0.477 |
| Other | 1.23 (0.95, 1.60) | 0.120 |
|  |  |  |
| **BCLC stage** |  |  |
| 0/A | 1 |  |
| B | 2.45 (1.54, 3.92) | <0.0001 |
| C | 2.27 (1.58, 3.26) | <0.0001 |
| D | 5.83 (3.62, 9.41) | <0.0001 |
|  |  |  |
| **ln(NLR)** | 1.98 (1.68, 2.35) | <0.0001 |

**LEGENDS TO SUPPLEMENTARY FIGURES**

**Supplementary Figure 1. Box plots of NLR at baseline versus last visit/death for each of the CLD and HCC groups.**

**Supplementary Figure 2. Kaplan- Meier survival curves according to CLD and HCC groups. Log-rank test, p-value <.0001.**
